# Physical Activity and Mediterranean Diet: A Focus on University Students’ Habits

**DOI:** 10.3390/nu17182951

**Published:** 2025-09-13

**Authors:** Vincenza Sansone, Silvia Angelillo, Giovanna Paduano, Gaia D’Antonio, Concetta Paola Pelullo, Gabriella Di Giuseppe

**Affiliations:** 1 Department of Experimental Medicine, University of Campania “Luigi Vanvitelli”, 80138 Naples, Italy; vincenza.sansone@unicampania.it (V.S.); silvia.angelillo@unipegaso.it (S.A.); giovanna.paduano@unicampania.it (G.P.); gaia.dantonio@unicampania.it (G.D.); 2 Department of Psychology and Health Sciences, Pegaso University, 80143 Naples, Italy; 3 Department of Medical, Human Movement and Well-Being Sciences, University of Naples “Parthenope”, 80133 Naples, Italy; concettapaola.pelullo@uniparthenope.it

**Keywords:** dietary habits, mediterranean diet, physical activity, prevention, survey, university students

## Abstract

**Background**: Physical inactivity and unhealthy dietary habits are among the major global public health concerns, contributing significantly to the increasing prevalence of non-communicable diseases. **Objectives**: The present study aimed to investigate the relationship between physical activity (PA) and dietary choices among undergraduate university students in Southern Italy. **Methods**: The cross-sectional survey was carried out through an anonymous web-based questionnaire. **Results**: Among the 500 university students who agreed to participate, only 3.4% of students reported regularly consuming five or more portions of fruits and vegetables per day, while 43.8% consumed 3–5 portions of starchy foods. Regarding fats, 31.2% reported consuming 2–3 portions of olive oil or butter per day. Men and those not having a health problem in the previous 12 months were less likely to adhere to the World Health Organization (WHO) recommendations on PA. Older students, those who consumed at least 5 meals per day, and those who acquired information from at least one source of information were more likely to adhere to the WHO recommendations on PA. **Conclusions**: Targeted initiatives promoting regular PA and healthy diets are essential to improving students’ health and well-being.

## 1. Introduction

Physical inactivity (PI) and unhealthy dietary habits are among the major global public health concerns contributing significantly to the increasing prevalence of obesity and related non-communicable diseases (NCDs) (such as cardiovascular disease, diabetes, and metabolic disorders) [1]. According to the World Health Organization (WHO), insufficient physical activity (PA) is one of the leading risk factors for morbidity and premature mortality worldwide. The WHO recommends at least 150–300 min of moderate-intensity aerobic activity per week, or at least 75–150 min of vigorous-intensity aerobic activity for adults to reduce the risk of chronic diseases and improve overall well-being, or an equivalent combination of both [2]. Despite strong evidence supporting these guidelines, a significant proportion of adults worldwide fails to meet the recommended levels of PA leading to widespread health implications [3].

In addition to PI, poor dietary choices contribute to the obesity epidemic with increasing consumption of energy-dense, ultra-processed foods high in saturated fats, refined sugars, and sodium [4,5]. In contrast, adherence to healthy dietary patterns, such as the Mediterranean Diet (MD), has been associated with a lower risk of obesity and chronic diseases [6]. The MD recognized by the WHO emphasizes a diet rich in fresh fruits, vegetables, whole grains, legumes, nuts, olive oil, and moderate consumption of fish and dairy. However, adherence to such dietary patterns is frequently unsatisfactory especially in younger people [7].

The Italian National Prevention Plan 2020–2025 encourages the importance of structured interventions to promote PA and healthy eating habits among young adults [8]. Several studies have shown that university students are particularly vulnerable to sedentary lifestyles, influenced by individual and social factors such as time, social support, and physical environment [9,10]. In addition, poor dietary habits are prevalent among this population and increase the risk of weight gain and metabolic imbalances [11,12].

It is well known that there is a strong correlation between healthy behaviors; in particular, there is a positive association between a healthy diet with PA [13], the number of meals consumed in a day [14], and less risky alcohol consumption [15] among university students. On the contrary, in several studies, there is an association between intensive PA and risky alcohol consumption [16,17], and this issue needs to be better explored to understand the reason for this phenomenon among younger, indeed, they could seek to compensate unhealthy behaviors (e.g., alcohol consumption) with healthy behaviors (e.g., PA) [18], and it is also emerged among adolescent students [19].

Despite the interest of international literature in these topics, there is insufficient evidence on how PA and MD are strictly interdependent, especially among the university student population preparing for adulthood.

The present study aimed to investigate the relationship between PA and dietary choices among undergraduate university students in Southern Italy.

## 2. Materials and Methods

### 2.1. Setting and Participants

The cross-sectional survey, which is part of a large observational study exploring youth lifestyle, was carried out through an anonymous web-based questionnaire developed with Google Forms^®^ [20]. The study reported was assessed following the STROBE guidelines for observational studies [21]. The data were collected between April and October 2023 among the target population of university students aged 18 to 30 attending Italian universities.

#### 2.1.1. Sample Size

The sample size was determined using a single population formula, assuming that 64% of people adhere to the WHO physical activity recommendations [22], using a confidence level of 95%, a margin of error of 5%, and considering a response rate of 95%. The sample size was estimated to be 355 participants. In order to increase the precision of the results, a larger non-probabilistic sample of university students was recruited.

#### 2.1.2. Data Collection

University students were recruited by sending an email to participate in the survey. The email contained a link to a private Telegram channel. Each participant, once entered into the Telegram channel, accessed an information sheet describing the aim of the study. Responders gave their informed consent before being involved in the survey for processing data by completing the questionnaire, and they were only allowed to respond once. No compensations or gifts were assigned to the respondents. The study protocol was approved by the Ethical Committee of the University of Campania “Luigi Vanvitelli” (No.14975/2023). The criteria of eligibility for the study were as follows: (i) age between 18 and 30 years, and (ii) enrolled in any Italian university. Students who declined to sign the informed consent were excluded from the study.

### 2.2. Survey Instrument

The self-administered questionnaire included five sections, and the minimum and maximum time to complete was 10 and 20 min, respectively. The first part included socio-demographic and anamnestic information, such as age, gender, university geographical area, degree course, underlying chronic medical diseases, and weight and height for Body Mass Index (BMI) calculation. The self-perceived health status was measured on a Likert-type scale ranging from 1 (not at all satisfied) to 10 (very much satisfied). The second section investigated PA levels using WHO guidelines on PA and sedentary behaviors. WHO recommends that adults accumulate at least 150 min of moderate-intensity PA or 75 min of vigorous-intensity PA per week, or an equivalent combination (2 min of moderate-intensity PA is equivalent to 1 min of vigorous-intensity PA) [2]. The third section investigated dietary habits asking the number of daily meals [23] and the adherence to all recommendations of the Italian Food Pyramid: water intake of 1.5 L per day; at least 5 portions of fruit and vegetables per day; 3–5 daily portions of starchy foods per day; 2–3 portions of oil or butter per day; 2–3 portions of dairy product or milk per day; 2 portions of protein sources per day; occasional consumption of snacks and sweets [24]. In the last part, participants were asked to indicate if they would like to obtain further information about prevention, with a “yes/no” answer.

Before starting the survey, a pilot study was conducted among 30 university students to ensure that items were clear and easy to answer. Questions were not modified, and the answers were not included in the final sample.

### 2.3. Statistical Analysis

Statistical analyses were performed with Stata software version 17 [25]. Firstly, descriptive analysis of the data was conducted to summarize the main characteristics of the sample, through frequency distribution and using numerical summary (mean) and dispersion (standard deviation, range). Second, the chi-square test, Fisher test, and the Student *t*-test were used to identify determinants associated with outcomes of interest. Then, a stepwise multivariate logistic regression model, with backward elimination, was designed for the following outcome: WHO PA recommendations (Model), which was recorded as 1 if the WHO PA recommendations were met and 0 if they were not. The following independent variables, which were judged to be potentially determinant of the outcome according to the literature and on clinical and behavioral plausibility, were selected: age in years (continuous), gender (male = 0; female = 1), university (Southern Italy = 1; Center Italy = 2; North Italy = 3), enrolled in degree course in Medicine and Health (no = 0; yes = 1), having an HCW in the family (no = 0; yes = 1), having at least one chronic medical condition (no = 0; yes = 1), self-perceived health status (continuous), having a partner (no = 0; yes = 1), having had a health problem in the previous 12 months (no = 0; yes = 1), smoking status (no = 0; yes = 1), alcohol use (no = 0; yes = 1), consumption of a correct number of meals per day (<5 = 0; ≥5 = 1), correct consumption of fruits and vegetables portions per day (no = 0; yes = 1), correct consumption of protein sources portion per day (no = 0; yes = 1), adequate daily water intake (no = 0; yes = 1), occasional consumption of snacks and sweets (no = 0; yes = 1), correct consumption of starchy foods per day (no = 0; yes = 1), correct consumption of oil or butter per day (no = 0; yes = 1), acquired information from at least one source (no = 0; yes = 1). Results are presented as Odds Ratio (ORs), and 95% confidence intervals (CIs) were calculated. All reported *p*-values are two-tailed, and a value < 0.05 is considered statistically significant.

## 3. Results

### 3.1. Socio-Demographic and Health-Related Characteristics of University Students

In total, 537 questionnaires were sent via email, and 500 university students agreed to participate, with a response rate of 93%. Table 1 displays an overview of university students’ socio-demographic and health-related characteristics.

More than three-fourths of the sample (77.1%) were female, and the ages of the students ranged from 19 to 30, with a mean age of 23.7 years. Participants attended a total of 51 Italian universities, 79.6% were from Southern Italy (n = 398), 7.6% Centre Italy (n = 38), and 12.8% Northern Italy (n = 64). The main fields of study were, specifically, Medicine and Health (33.4%), Arts and Humanities (9.2%), Language (2.8%), Business and Social Sciences (33%), Science & Technology (14.8%), and Engineering (6.8%). One third of the respondents (31.6%) have an HCW in the family, and 14% of university students had at least one chronic medical condition. Regarding weight status, 73% of the sample was classified as having a healthy weight, while 5.3% was underweight, and 21.7% was overweight/obese. Moreover, respondents reported that their self-perceived health status, measured on a 10-point Likert-type scale, had an average of 7.7 (SD ± 1.4). More than one-third of the respondents (43%) had had at least one health problem in the previous 12 months. Concerning smoking status, 276 young adults (55.2%) had never smoked, 15.6% and 29.2% were former and current smokers, respectively. Finally, only 12.4% had never consumed alcoholic beverages.

### 3.2. Behaviors Regarding Dietary Habits and Physical Activity

Based on the questionnaire responses, 7.4% of students reported consuming two meals per day, 36.4% consumed three meals, 30.8% consumed four meals, and 25.4% regularly consumed five or more meals per day. The descriptive analysis for the adequacy of food consumption is detailed in Table 2.

The reported food items were categorized according to the levels of the Italian Food Pyramid. Only 3.4% of students reported regularly consuming five or more portions of fruits and vegetables per day, while 43.8% consumed 3–5 portions of starchy foods per day. Regarding fats, 31.2% reported consuming 2–3 portions of olive oil or butter per day. For dairy products, 16% reported consuming 2–3 portions per day. Protein sources, including meat, fish, legumes, and eggs, were consumed in two daily portions by 22% of students. Additionally, 57.4% of students reported occasionally consuming snacks and sweets. Moreover, 52.6% of young adults had an adequate daily water intake. It is noteworthy that no student adhered to all the recommendations outlined by the Italian Food Pyramid across all dietary. Moreover, when participants were asked about their sitting time, young adults spent an average of 359.6 min a day seated (SD ± 200) during working days and an average of 305.1 min a day seated (SD ± 194.6) during non-working days. More than one-fourth of the participants (27.2%) reported engaging in vigorous PA in the past 7 days, with at least 75 min per week. Merely 10.5% reported engaging in moderate PA during the same period, with at least 150 min per week. Two-thirds (64.1%) of university students meet the WHO PA recommendation. Table 3 displays the results of the multivariate logistic regression model that identifies several factors associated with meeting WHO PA recommendations among university students. Men (OR = 0.41; 95% CI = 0.23–0.72) and those not having a health problem in the previous 12 months (OR = 0.60; 95% CI = 0.41–0.92) were less likely to adhere to the WHO recommendations on PA. While older (OR = 1.09; 95% CI = 1.00–1.19), those who consumed at least 5 meals per day (OR = 2.08; 95% CI = 1.25–3.48), and those who acquired information from at least one source (OR = 3.20; 95% CI = 1.19–8.62) were more likely to adhere to the WHO recommendations on PA.

## 4. Discussion

Our study provides an overview of the dietary habits and PA levels, of a sample of Italian university students, offering insights of their lifestyle, and highlighting several areas of concern, particularly about poor adherence to dietary guidelines and WHO PA recommendations.

Regarding dietary habits, our findings show a substantial gap between guidelines and the current food intake of participants. Indeed, although the majority of them consumed three or more meals, only 3.4% met the recommended intake of at least five portions of fruits and vegetables daily; this value is similar to those reported in other studies [26,27], where low fruit and vegetable consumption is common among university students. Furthermore, adherence to the Italian Food Pyramid recommendations was generally poor overall. This result is particularly concerning, and it reflects a trend already identified in the literature, in which university students often develop unhealthy eating habits due to different factors, such as time, financial support, and lack of nutritional awareness [28,29]. This also aligns with findings from Liu et al. (2024), who reported that suboptimal dietary patterns among young adults were also significantly associated with metabolic syndrome [11]. Similarly, another study conducted in China emphasized how the accumulation of bad dietary habits increases the risk of metabolic syndrome development, reinforcing the long-term implications of an unhealthy diet in this population [12].

Moreover, while starchy foods and fats showed moderate levels of adherence, intake of milk or dairy products and protein sources remained suboptimal for many students. This behavior should be improved because an inadequate consumption of these products compromises bone health, which is dependent on adequate calcium and protein intake during young adulthood. Additionally, when we investigated the water intake, the respondents reported it as adequate by just over half of the sample (52.6%). These findings are consistent with other similar studies indicating that young adults, particularly university students, frequently adopt unhealthy eating habits, with potential long-term consequences [9,10,12,30].

Interestingly, in our sample, meal frequency was positively associated with PA adherence. Indeed, university students who consumed five or more meals per day were significantly more likely to adhere to the WHO recommendations on PA. This finding is in line with previous studies that showed that regular meals are associated with healthier lifestyles overall [31,32].

With regard to PA, nearly half of the students engaged in vigorous activity, and approximately 70% reported engaging in moderate PA during the same period of the survey. While this value should be encouraging, the mean sitting time remained high both during working and non-working days, showing that sedentary behaviors are still frequent. This is consistent with other university cohorts globally [33] and with Cepková et al. (2023) [9], who found that prolonged sedentary behavior in university students negatively affected spinal health, particularly in the thoracic region for men and the lumbar region for women. Indeed, it is well recognized that prolonged sedentary behavior is an independent health risk factor, even in physically active individuals, highlighting the need for interventions that target both movement and sitting time.

Moreover, our multivariate logistic regression analysis revealed important predictors of adherence to the WHO PA recommendations. Specifically, older students, those who consumed at least five meals per day, and those who acquired information from at least one source of information were more likely to adhere to the WHO recommendations on PA. These results are similar to previous published studies, suggesting that health literacy is positively associated with PA engagement [34,35].

Conversely, male students and those not having a health problem in the previous 12 months were less likely to meet the recommendations on PA. This latter result may suggest that personal health experiences act as motivators for adopting more active lifestyles. The association between male gender and lower compliance with PA recommendations is somewhat unexpected; indeed, previous studies have often shown that women perform less PA than men, particularly during college [36]. Despite this, another study has also highlighted how, in a similar population, women reported higher exercise than men during their university years [37]. This element requires more in-depth analysis to understand how to involve women in participating in PA. This discrepancy might be explained because women in this cohort, particularly those enrolled in a degree course in Medicine and Health, may have higher motivation or awareness regarding the importance of PA and its implications on health.

In light of the results obtained, it is clear that public health interventions aimed at improving the acquisition of information among university students regarding WHO recommendations on PA are needed. The university setting appears to be an optimal context to spread the knowledge regarding lifestyles and to promote intervention to provide accessible and low-cost PA, such as participation in activity programs integrated in the university curriculum, find dedicated spaces for PA on the campus [10,38]. Additionally, variety of food items provided by university canteens, organizing spaces for students to warm up and eat their homemade foods, offering discounts at supermarkets, and providing information about nutrition and a healthy diet could be important strategies to guide students’ healthy choices [39]. In particular, women and younger people should be majorly involved in information campaigns in order to improve their adherence to the WHO recommendations and establish correct behaviors to pursue them into adulthood and prevent NCDs.

### Limitation

This study has several limitations that should be considered in the interpretation of the results. First, the cross-sectional design does not allow for determining the causal association between the different predictors and the outcomes of interest. Second, self-reported data may introduce recall and social desirability biases, particularly for healthy behaviors, such as dietary habits and PA, which may not accurately reflect their long-term habits. However, participants were assured that their answers were completely anonymous, and this may have increased honesty in completing the questionnaire. Third, the use of Telegram could have had an impact on the participation since not all students use this platform. Finally, our sample is based only on a non-probabilistic sampling approach, and this may increase the risk of selection bias and limit the representativeness of all university students in Italy and of people with the same age who do not attend university. Indeed, in Italy, less than half of the young attend a university course [40], the majority are women [41]. Despite these limitations, the strengths of this study were the fact that the sample was large and properly selected and the high response rate.

## 5. Conclusions

This study found low adherence to both dietary guidelines and WHO PA recommendations among university students in Italy. However, all these findings highlight the potential role of dietary regularity and information access as key facilitators in promoting PA engagement. Providing clear, evidence-based dietary information can help students to make conscious lifestyle choices, reinforcing healthy behaviors such as regular PA. Therefore, in light of these results, there is a need for targeted educational programs to improve healthy eating habits and regular PA within this population. Such initiatives could be useful as preventive measures to raise awareness about the benefits of healthy lifestyles and to promote gradual changes in behavior among university students.

## Figures and Tables

**Table 1:** Socio-demographic and health-related characteristics of university students.

			Adherence to the WHO Physical Activity Recommendations			
Socio-Demographic and Health-Related Characteristics	Total (n: 500)		No (n: 174, 35.9%)		Yes (n: 310, 64.1%)	
	N	%	N	%	N	%
**Age, (years)**	23.7 ± 2.6 (19–30) *		*t*-test (482) = −0.94, *p* = 0.3441			
**Gender ^a^**						
Male	113	22.9	25	22.9	84	77.1
Female	381	77.1	146	39.5	224	60.5
			*χ*^2^ = 10, *df* = 1, *p* = 0.002			
**University ^a^**						
Southern Italy	398	79.6	127	33.2	256	66.8
Centre Italy	38	7.6	18	47.4	20	52.6
North Italy	64	12.8	29	46	34	54
			*χ*^2^ = 6.2, *df* = 2, *p* = 0.044			
**Degree Course in Medicine and Health ^a^**						
No	333	66.6	119	37.1	202	62.9
Yes	167	33.4	55	33.7	108	66.3
			*χ*^2^ = 0.52, *df* = 1, *p* = 0.471			
**Have an HCW in the family ^a^**						
No	342	68.4	122	36.9	209	63.1
Yes	158	31.6	52	34	101	66
			*χ*^2^ = 0.37, *df* = 1, *p* = 0.541			
**Chronic medical condition ^a^**						
No	430	86	152	36.6	263	63.4
Yes	70	14	22	31.9	47	68.1
			*χ*^2^ = 0.58, *df* = 1, *p* = 0.447			
**Body Mass Index Category ^a^**	22.9 ± 3.6 (16–46.9) *		*t*-test (487) = −0.94, *p* = 0.349			
Underweight	26	5.3	11	44	14	56
Healthy weight	357	73	128	36.8	220	63.2
Overweight-Obese	106	21.7	31	31	69	69
			*χ*^2^ = 1.87, *df* = 2, *p* = 0.392			
**Self-perceived health status**	7.7 ± 1.4 (3–10) *		*t*-test (482) = −1.1, *p* = 0.272			
**Health problem in the previous 12 months ^a^**						
No	285	57	93	33.8	182	66.2
Yes	215	43	81	38.8	128	61.2
			*χ*^2^ = 1.25, *df* = 1, *p* = 0.262			
**Smoking status ^a^**						
Never smoker	276	55.2	97	36.2	171	63.8
Former smoker	78	15.6	25	33.3	50	66.7
Current smoker	146	29.2	52	36.9	89	63.1
			*χ*^2^ = 0.28, *df* = 2, *p* = 0.868			
**Alcohol consumption ^a^**						
No	61	12.4	28	47.5	31	52.5
Yes	430	87.6	143	34.4	273	65.6
			*χ*^2^ = 3.84, *df* = 1, *p* = 0.050			
**Minutes spent sitting during working days ^a^**	359.6 ± 200 (0–900) *		*t*-test (400) = 0.95, *p* = 0.342			
**Minutes spent sitting on non-working days ^a^**	305.1 ± 194.6 (0–900) *		*t*-test (426) = 0.99, *p* = 0.3224			
**At least 5 daily meals ^a^**						
No	373	74.6	142	39	222	61
Yes	127	25.4	32	26.7	88	73.3
			*χ*^2^ = 5.97, *df* = 1, *p* = 0.015			
**At least 5 daily portions of fruit and vegetables ^a^**						
No	483	96.6	172	36.8	296	63.2
Yes	17	3.4	2	12.5	14	87.5
			F test = 0.062, *df* = 1			
**3–5 daily portions of starchy foods ^a^**						
No	281	56.2	96	35.3	176	64.7
Yes	219	43.8	78	36.8	134	63.2
			*χ*^2^ = 0.12, *df* = 1, *p* = 0.733			
**2–3 daily portions of oil or butter ^a^**						
No	344	68.8	121	36.7	209	63.3
Yes	156	31.2	53	34.4	101	65.6
			*χ*^2^ = 0.23, *df* = 1, *p* = 0.631			
**2–3 daily portions of dairy product or milk ^a^**						
No	420	84	152	37.3	256	62.7
Yes	80	16	22	28.9	54	71.1
			*χ*^2^ = 1.92, *df* = 1, *p* = 0.166			
**2 daily portions of protein sources ^a^**						
No	390	78	140	37	238	63
Yes	110	22	34	32.1	72	67.9
			*χ*^2^ = 0.89, *df* = 1, *p* = 0.347			
**Correct daily water intake ^a^**						
No	237	47.4	89	39.7	135	60.3
Yes	263	52.6	85	32.7	175	67.3
			*χ*^2^ = 2.59, *df* = 1, *p* = 0.108			
**Occasional consumption of snacks and sweets ^a^**						
No	213	42.6	80	38.8	126	61.2
Yes	287	57.4	94	33.8	184	66.2
			*χ*^2^ = 1.30, *df* = 1, *p* = 0.255			
**Use of sources on information ^a^**						
Nothing	27	5.4	13	56.5	10	43.5
At least one	473	94.6	161	34.9	300	65.1
			*χ*^2^ = 4.44, *df* = 1, *p* = 0.035			

* Mean ± Standard Deviation (range); ^a^ Number of each item may not add up to the total number of the study population due to missing values. WHO: World Health Organization; HCW: Healthcare Worker.

**Table 2:** Adequacy of food consumption according to the levels of the Italian Food Pyramid (n: 500).

Levels of the Italian Food Pyramid	Recommended Portions	N (%) of Adequate Consumption
Fruit and vegetables	5 or more per day	17 (3.4)
Starchy foods	3–5 per day	219 (43.8)
Oil or butter	2–3 per day	156 (31.2)
Dairy product or milk	2–3 per day	80 (16)
Protein sources (meat, fish, legumes, eggs)	2 per day	110 (22)
Snacks and sweets	Occasionally	287 (57.4)

**Table 3:** Results of the multivariate logistic regression analysis about students who adhere to the WHO recommendations on PA.

Variables	OR	95% CI	*p*
Model. Students who adhere to the WHO recommendations on PA Log likelihood = −254.61519, *χ*^2^ = 45.90 (*df* = 9), *p* < 0.0001, No. of obs = 426, Pseudo-R^2^ = 0.0827			
Male	0.41	0.23–0.72	0.002
Consumption of at least 5 meals per day	2.08	1.25–3.48	0.005
Having not a health problem in the previous 12 months	0.60	0.41–0.92	0.019
Having acquired information from at least one source	3.20	1.19–8.62	0.021
Older	1.09	1.00–1.19	0.048
Correct consumption of fruits and vegetables portions per day	6.99	0.85–57.35	0.070
Adequate daily water intake	1.45	0.95–2.20	0.085
Alcohol use	1.73	0.90–3.33	0.101
University			
North Italy	1.00 *		
Southern Italy	1.47	0.87–2.46	0.146
Centre Italy	Backward elimination		

WHO: World Health Organization; PA: Physical Activity; * Reference category.

## Data Availability

The data presented in this study are available upon request from the corresponding author due to ethical restrictions.
